# Opportunities and Challenges for Lignin Valorization in Food Packaging, Antimicrobial, and Agricultural Applications

**DOI:** 10.1021/acs.biomac.2c01385

**Published:** 2023-02-06

**Authors:** Alice Boarino, Harm-Anton Klok

**Affiliations:** † Institut des Matériaux and Institut des Sciences et Ingénierie Chimiques, Laboratoire des Polymères, École Polytechnique Fédérale de Lausanne (EPFL), Station 12, CH-1015 Lausanne, Switzerland

## Abstract

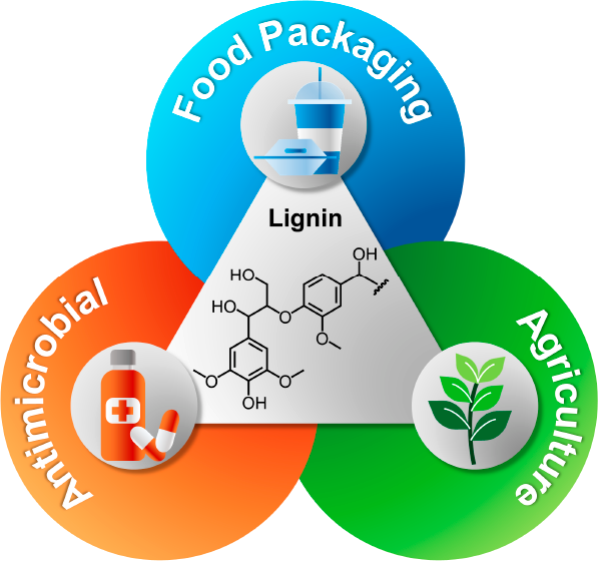

The exploration of renewable resources is essential to help transition toward a more sustainable materials economy. The valorization of lignin can be a key component of this transition. Lignin is an aromatic polymer that constitutes approximately one-third of the total lignocellulosic biomass and is isolated in huge quantities as a waste material of biofuel and paper production. About 98% of the 100 million tons of lignin produced each year is simply burned as low-value fuel, so this renewable polymer is widely available at very low cost. Lignin has valuable properties that make it a promising material for numerous applications, but it is far from being fully exploited. The aim of this Perspective is to highlight opportunities and challenges for the use of lignin-based materials in food packaging, antimicrobial, and agricultural applications. In the first part, the ongoing research and the possible future developments for the use of lignin as an additive to improve mechanical, gas and UV barrier, and antioxidant properties of food packaging items will be treated. Second, the application of lignin as an antimicrobial agent will be discussed to elaborate on the activity of lignin against bacteria, fungi, and viruses. Finally, the use of lignin in agriculture will be presented by focusing on the application of lignin as fertilizer.

## 1 Introduction

**Figure 1:**
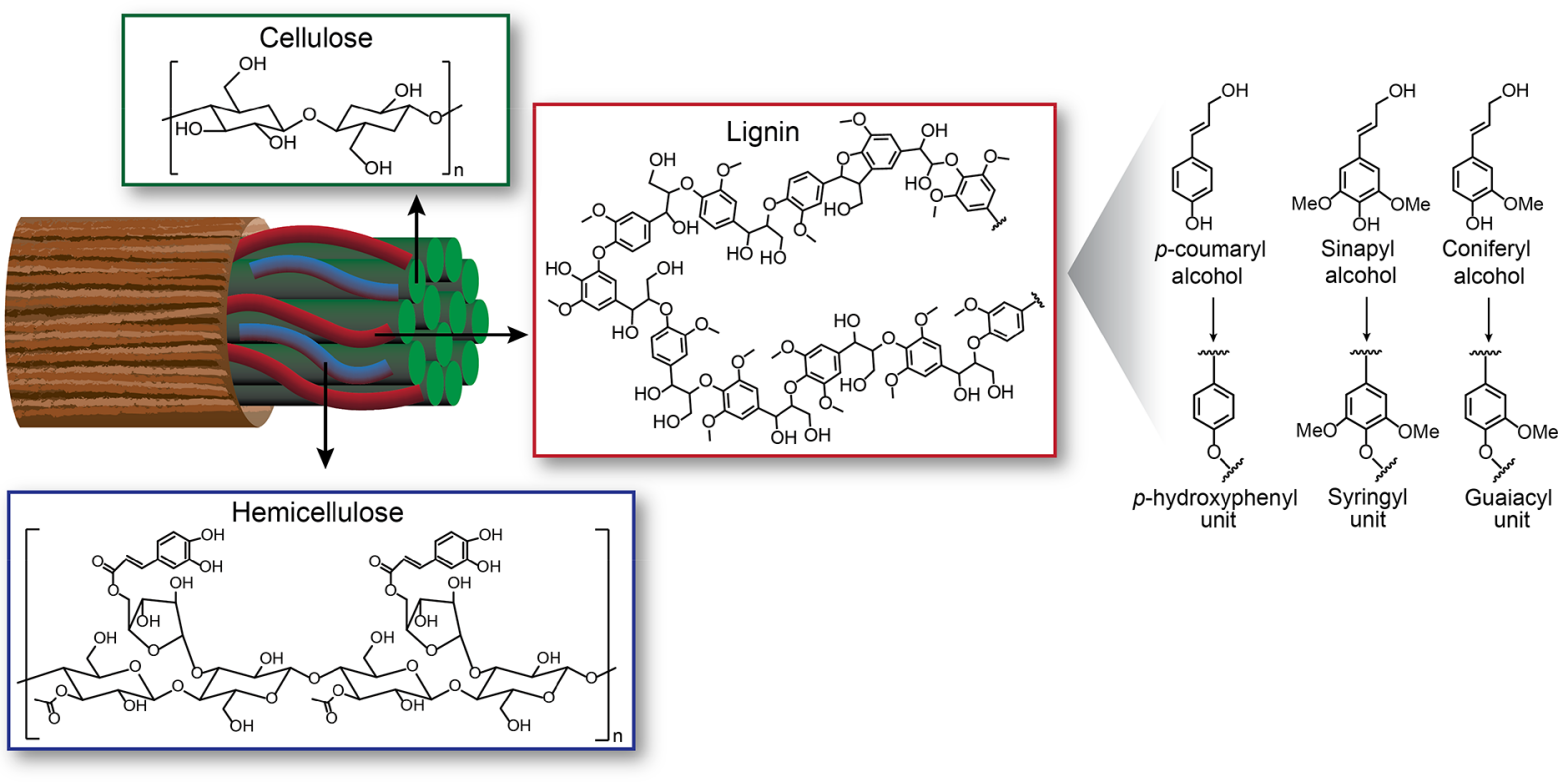
Representation of cellulose, hemicellulose, lignin, and lignin structural units.

The world dependence on and excessive use of fossil fuels have led to climate change, which has forced researchers and industries to focus their attention on the exploration of renewable and green alternatives to oil, natural gas, and coal. First-generation biorefineries address this issue through the fermentation of corn, sugar cane, and wheat to obtain bioethanol^1^ and the transesterification of rapeseed and soybean oil to produce biodiesel.^2^ Although these are well-established processes to generate green energy, their sustainability is still under debate because they utilize edible crops and, thus, compete with food production. To avoid, for example, deforestation to free the extensive land that these crops need and a potential increase of food price, a new generation of biorefineries is being developed that aim to utilize nonedible lignocellulosic biomass.^3,4^ In particular, the valorization of lignin, one of the main biomass components, holds great promise for contributing to the successful development of future biorefineries.^5,6^ Lignin is a cross-linked aromatic heteropolymer, which, together with cellulose and hemicellulose, is found in the plant cell wall (Figure 1) where it provides mechanical support and protection against pathogens.^7^ Lignin makes up 15–35% of lignocellulosic biomass, and ∼100 million tons of this biopolymer are yearly isolated as waste material from the paper and bioethanol industry.^8^ Less than 2% of this enormous quantity is currently commercialized as low-value products, such as surfactants and adhesives, while the rest is mainly burned.^5,8^ The application of this underutilized biopolymer is, hence, attractive from both a sustainability and economic point of view.

Lignin biosynthesis takes place via oxidative radical polymerization of coniferyl, sinapyl, and *p*-coumaryl alcohol^9,10^ that is triggered by a series of enzymes, which includes laccases and peroxidases as key players.^11^ Once incorporated in the lignin polymer, these structural units are referred to as guaiacyl, syringyl, and *p*-hydroxyphenyl units, respectively.^10,12−14^ The structure of lignin and these different building blocks is shown in Figure 1. Since lignin is generated via coupling reactions between phenolic radicals, the molecular weight distribution and composition of this biopolymer is very heterogeneous and can widely vary depending on the plant species.^14,15^

Lignin can be industrially isolated from various natural sources, such as woody biomass, agricultural residues, and energy crops.^5^ There are four main biorefinery processes used for lignin extraction: sulfite, soda, kraft, and organosolv. As summarized in Table 1, they present different features and afford technical lignins with different properties. Generally speaking, they apply high temperature and/or highly acid or basic conditions that cleave the lignin ether bonds to result in the formation of oligomers containing stable C–C bonds, which cannot be further modified and, thus, hinder lignin depolymerization into individual monomers. A number of research groups have developed methods to avoid the formation of C–C bonds during lignin extraction. This allows the depolymerization of lignin to produce a wide number of aromatic monomers.^9,16,17^ As a consequence, the valorization of lignin can both involve the use and application of the whole polymer, as well as the exploration of opportunities for the low-molecular-weight oligomers that are obtained via lignin depolymerization.^5^

**Table 1:** Overview of Technical Lignin Extraction Processes, And Solubility, Weight-Average Molecular Weight (*M*_w_), Dispersity (*Đ*), and Impurities in the Different Types of Lignins

lignin type	extraction process^18,19^	solubility^20,21^	M_w_ (kDa)^20,21^	Đ^21,22^	impurities^20^
kraft lignin	170 °C, NaOH, Na_2_S	aqueous media pH > 10	0.1–3	2.5–3.5	sulfur
lignosulfonates	140 °C, SO_2_, Na^+^/Ca^+^/Mg^+^/NH_4_^+^	water	20–50	6.0–8.0	sulfur
organosolv lignin	150–200 °C, acetic acid/formic acid/organic solvents	organic solvents	0.5–4	1.3–4.0	carbohydrates and ash
soda lignin	150–170 °C, NaOH	aqueous media pH > 10	0.8–3	2.5–3.5	carbohydrates and ash

Besides being both economically and environmentally friendly, lignin also presents intrinsic properties that make it an attractive material to be used in a wide range of applications, as reported in a number of review articles.^12,13,23,24^ The aim of this Perspective is to highlight opportunities and challenges for the use of lignin-based materials in three, which we believe are, important and promising areas of application, namely food packaging, antimicrobial applications, and agriculture. Herein, we not only give an overview of the role of lignin in these three application fields, but we also highlight the challenges and problems that still need to be addressed and we provide a forward-looking perspective on the possible future developments on this topic.

## 2 Lignin in Food Packaging

**Figure 2:**
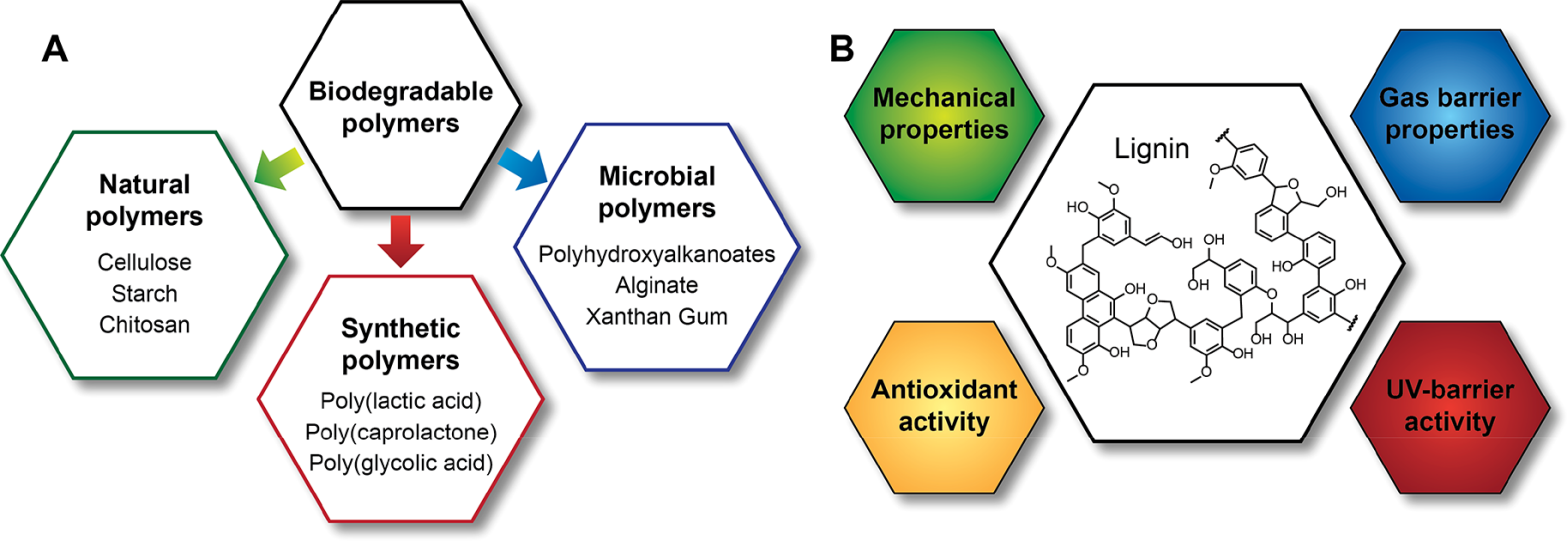
(A) Biodegradable polymers utilized in food packaging. (B) Properties that lignin incorporation can affect, when incorporated in a biodegradable polymer film.

Every year, 140 million tons of plastic are produced and utilized as packaging materials.^25,26^ Around 40% of this is for food packaging, where it is mostly designed to be single-use and not recycled. Food and food packaging currently are also responsible for almost half of the total municipal solid waste.^27^ Most of the polymers used for food packaging are nondegradable oil-derived materials, such as poly(ethylene terephthalate) (PET), polyethylene (PE), polypropylene (PP), polyvinyl chloride (PVC), and polystyrene (PS).^28,29^ A number of strategies are possible to reduce the use of petroleum-based resources and prevent the accumulation of discarded materials in the environment. One possibility is the implementation of food storage systems to prolong the food shelf life. Another approach is to substitute conventionally used polymers with biodegradable alternatives.^30^ Biodegradable polymers, which can be decomposed into CH_4_, CO_2_, and H_2_O by microorganisms, can be classified according to their sources into natural, microbial, and synthetic polymers (Figure 2A). It is important to highlight that the end-of-life management of these materials, such as industrial composting or home composting for some of them, remains as important as in the case of the oil-derived ones.^31−33^

Although the polymers that are highlighted in Figure 2A can be used to substitute non-biodegradable plastics and reduce the environmental impact, they generally have only moderate mechanical and barrier properties, and often are more expensive than the commonly utilized materials.^28^ For these reasons, they constitute only 1% of the plastics utilized for food packaging.^30^ To improve the market expansion of the materials shown in Figure 2A, the performance of these bioplastics needs to be improved. The introduction of lignin as a filler for biodegradable plastics is one way to achieve this goal. Lignin incorporation can modify the mechanical and gas barrier properties of food packaging films, and also provide the packaging material with properties such as antioxidant and UV-barrier activity (Figure 2B). Lignin can be incorporated either by blending free lignin with the polymer of interest or, alternatively, by using lignin nanoparticles. Table 2 lists examples of studies that have used lignin as a filler in biodegradable polymer films.

**Table 2:**
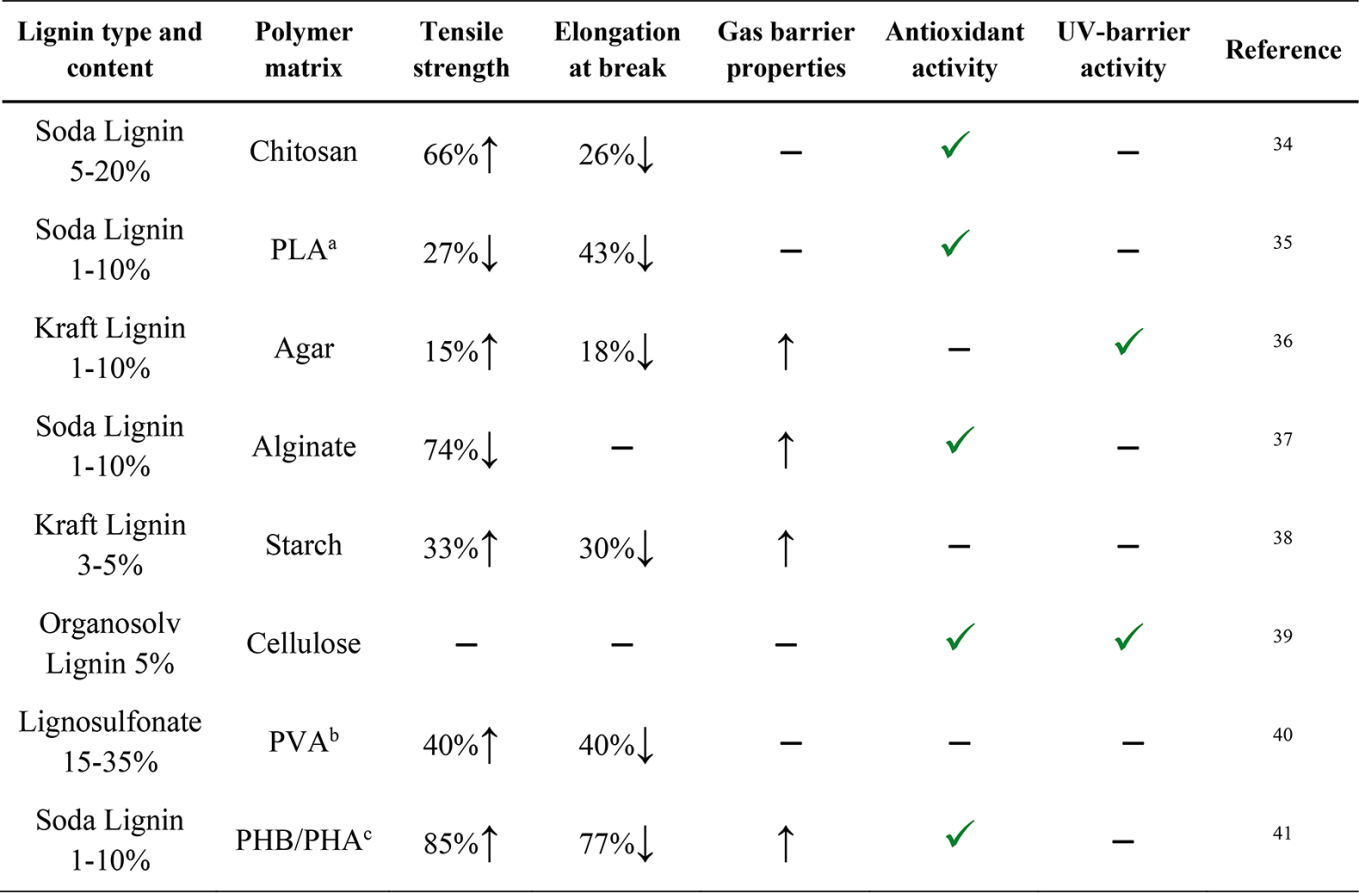
Examples of Studies Where Lignin Was Incorporated into Biodegradable Polymers and the Effect of Lignin Incorporation on Tensile Strength, Elongation at Break, Gas Barrier Properties, and Antioxidant and UV-Barrier Activity of the Resulting Composite Films^34−41^

a PLA = poly(lactic acid).

b PVA = poly(vinyl alcohol).

c PHB/PHA = poly(3-hydroxybutyrate)/polyhydroxyalkanoates.

In addition to blending free lignin, this biopolymer can also be incorporated in food packaging films in the form of nanoparticles. Lignin nanoparticles can be synthesized by different methods, such as precipitation via solvent or pH exchange, self-assembly, microwave assistance, ultrasonication, and aerosol processing.^42,43^ These particles can be incorporated in a matrix to prepare nanocomposites.^43,44^ The main advantage of using nanoparticles is that they present a high surface-area-to-volume ratio. There are some examples, in which lignin nanoparticles have been incorporated in a biodegradable polymer matrix to form nanocomposites that could potentially be used for food packaging (Table 3). As Table 3 indicates, typically only a small amount of lignin nanoparticles is incorporated in the polymer matrix (max 3%). This is because, at higher nanoparticle contents, the nanoparticles aggregate to form larger clusters. This is caused by the poor compatibility between the aromatic cross-linked lignin and the polymer matrix. This obstacle can be overcome by surface modification of the lignin nanoparticles in order to increase their compatibility with the surrounding matrix. In one example, this was accomplished by etherification of the surface of lignin nanoparticles with citric acid. In this way, 10 wt % of lignin nanoparticles could be incorporated inside a PVA film.^45^ Another approach involves grafting polymer chains from the particle surface. Ring-opening polymerization of lactide, for example, has been used to generate PLA-modified lignin nanoparticles that could be incorporated in a well-dispersed fashion to generate PLA films that contained up to 10 wt % lignin nanoparticles.^46^

**Table 3:**
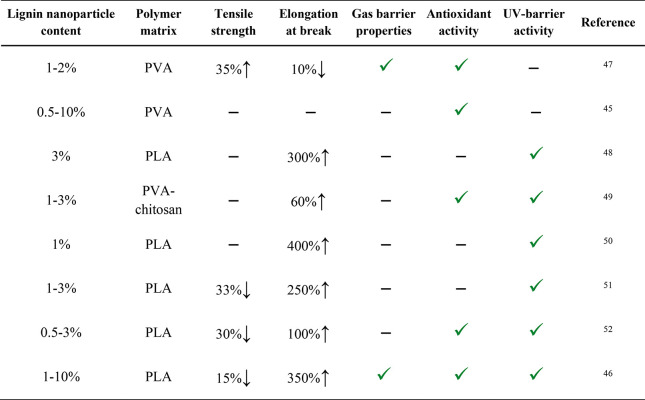
Examples of Studies Where Lignin Nanoparticles Were Incorporated into Biodegradable Polymers and the Effect of Lignin Incorporation on the Tensile Strength, Elongation at Break, Gas Barrier Properties, and Antioxidant and UV-Barrier Activity of the Resulting Films^47−52^

As mentioned above, the addition of lignin is attractive because it provides a way to improve mechanical and gas barrier properties, as well as the antioxidant activity and UV-barrier properties of food packaging materials. When lignin is incorporated in a polymer film, the mechanical properties can change in different ways. As Tables 2 and 3 highlight, the tensile strength and elongation at break can increase, decrease, or remain unchanged, depending on the polymer matrix and the type of lignin. The effect of lignin incorporation is, thus, film specific, but the take home message from the literature is that, overall, the compatibility between the lignin filler and the polymer matrix defines the mechanical properties of the film.^13^ Generally, a more efficient lignin dispersion and compatibilization lead to better mechanical properties. Possible ways to improve the compatibility between lignin and the polymer matrix and to avoid phase separation are lignin esterification,^53−58^ the use of cross-linkers,^59−61^ and polymer surface modification.^45,46,62,63^

Lignin incorporation can reduce the oxygen and water vapor transmission, particularly when films are made from hydrophilic materials, such as alginate^37^ and starch.^38^ This is not only because of the overall hydrophobic nature of lignin but also because of the interaction of lignin and film matrix. Once incorporated in the film, lignin interacts with the hydrophilic groups of the biopolymer, thereby reducing their affinity to water and oxygen molecules.^13^

Oxidation of lipids and proteins inside food is one of the main reasons for food deterioration, and it affects food appearance, taste, and smell and can lead to the generation of toxic aldehydes.^64^ Antioxidant compounds, which act as radical scavengers and delay radical oxidative processes, can be incorporated in packaging materials to prevent food oxidation. Typical examples of antioxidants are butylated hydroxyanisole and butylated hydroxytoluene.^65^ Although they are very efficient in hindering food oxidation, these compounds can generate benzoic acid, nitrates, and sulphites, which can cause allergies and may have other side effects on human health.^66,67^ Recently, the interest in greener and safer natural antioxidants has, therefore, increased. Lignin is an efficient antioxidant and a promising alternative for the mentioned synthetic compounds.^35,68,69^ The antioxidant activity of lignin is due to the presence of phenols in its structure, which can act as radical scavengers. A number of studies have verified that a higher phenol content, lower molecular weight, and narrower dispersities correlate with a higher antioxidant activity of lignin.^70−73^

The presence of chromophores, such as carbonyl and conjugated phenol groups, inside the lignin structure enables this polymer to absorb light in the UV range (200–400 nm).^24,74^ This is a further advantage of using lignin fillers in food packaging because they help to protect food from UV irradiation. It is important to consider that the UV protection provided by lignin comes with a loss of visible transparency of the polymer film because of the brown color of lignin. Visible transparency is an important factor for food packaging because customers generally desire to see the product inside the packaging. It is, hence, always important to optimize the lignin content and distribution inside the polymer film in order to find a material composition where the film is protecting the food from UV irradiation but also allowing the product to be visually seen.

### Challenges and Future Perspectives for the Use of Lignin in Food Packaging Applications

Lignin can be incorporated into biodegradable polymer films to improve their performance in food packaging. The addition of lignin can enhance mechanical and gas barrier properties, two of the main weaknesses of biodegradable polymers, and provide them with antioxidant and UV barrier properties, which are of major importance for food preservation. Among the challenges for the preparation of such blend materials is the compatibility between the polymer matrix, often made of linear aliphatic polymer chains, and the aromatic cross-linked structure of lignin. This leads to phase separation and heterogeneity inside the film, which limits the performance of the final product. The same applies for the preparation of nanocomposites where lignin nanoparticles are incorporated in the polymer film. Only very small amounts of nanoparticles have been introduced into such films, while a higher particle content could not be achieved without aggregation and phase separation. The functionalization of lignin and of lignin nanoparticles to improve their affinity with the polymer matrix is, thus, key to achieve an efficient dispersion of lignin in the final film. Another important issue that still needs to be addressed regarding the use of lignin in food packaging is safety. Studies about the interaction of lignin with the packaged food, as well as in vivo digestion, are to date very preliminary and will require additional investigation.

The design of sustainable food packaging items must take into consideration the end-of-life management of the final product. Most of the food packaging items are disposed of by landfilling, and are not recycled because of the presence of additives, as well as food contamination that can be challenging to separate. Landfilling results in the occupation of large amounts of space and the production of greenhouse gases, whereas composting is a valid alternative end-of-life treatment.^75^ The American Society for Testing and Materials (ASTM) defines a plastic as compostable when it “undergoes degradation by biological processes during composting to yield carbon dioxide, water, inorganic compounds, and biomass at a rate consistent with other known compostable materials and that leaves no visible, distinguishable, or toxic residue.”^76^ This is a subgroup of biodegradable plastics, which are instead defined as “plastic in which the degradation results from the action of naturally-occurring micro-organisms such as bacteria, fungi, and algae.”^76^ Therefore, not all biodegradable plastics are compostable. Lignin is efficiently biodegraded by white-rot fungi and various types of bacteria,^77,78^ but the degradation of lignin under composting conditions commonly used to dispose of food packaging items is incomplete and inefficient.^79^ Moreover, the properties introduced by the addition of lignin in a polymer matrix, such as improved gas barrier, decreased water permeability, and increased hydrophobicity, can reduce the material degradability in the composting conditions. Additional attention should be placed on studying how the introduction of lignin influences the compostability of the final product because this parameter is often not considered in the published studies.

## 3 Lignin as Antimicrobial Agent

**Figure 3:**
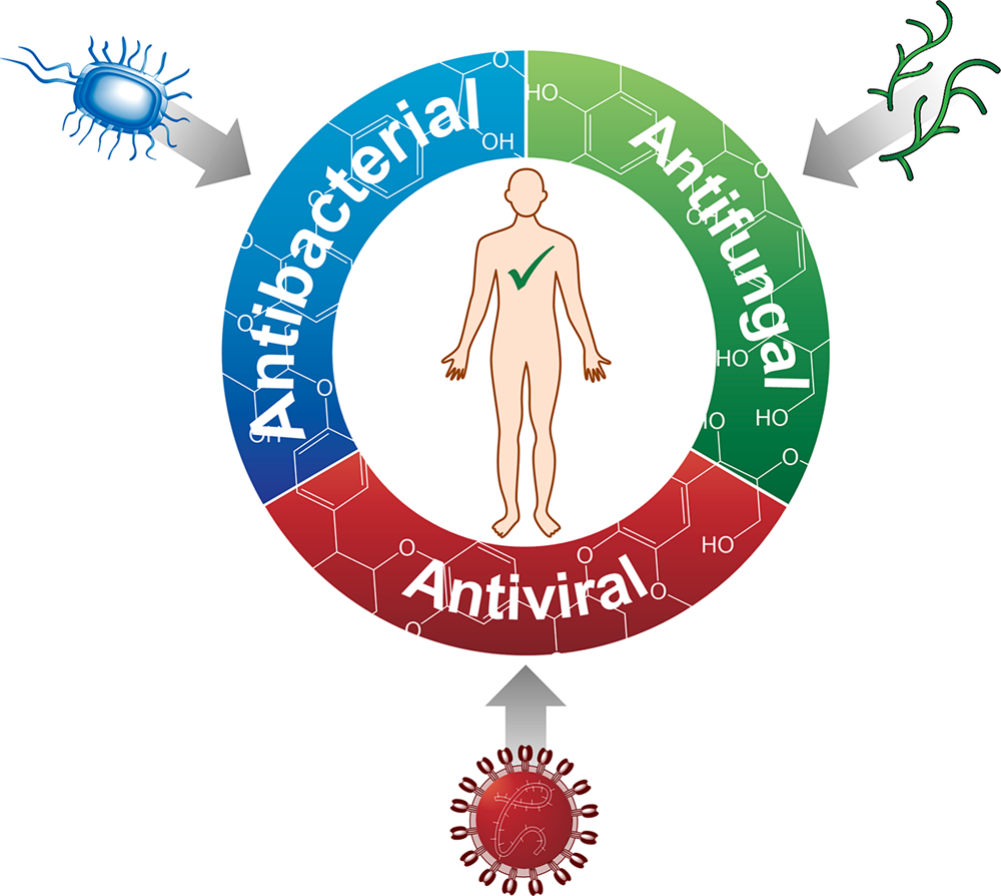
Antimicrobial activity of lignin.

Some bioactive compounds extracted from plants can be used as antimicrobial agents to inhibit the harmful activity of bacteria, fungi, and viruses. Common examples are polyphenols, amino acids, terpenoids, flavonoids, and tannins, which not only are very interesting for their biological activity but also for their biocompatibility, renewability, and biodegradability.^12,80^ Most of these compounds, however, are found in very small quantities in plants and typically require complex extraction processes to be isolated. Lignin has recently attracted much attention since it is cheap and accessible and also shows interesting biological activities. The antimicrobial activity of lignin derives from its natural ability to protect a plant from pathogens.^7^ Inside the plant, lignin can preserve carbohydrates from degradation by suppressing the attack of bacteria and fungi.^12^ Technical lignins isolated from lignocellulosic biomass have been involved in biological and medical studies as an antibacterial, antifungal, and antiviral agent (Figure 3). Table 4 presents several selected examples of studies that have investigated the antimicrobial activity of various lignins in solution.

**Table 4:** Examples of Studies That Have Investigated the Antimicrobial Properties of Lignin in Solution

lignin type	solvent	conc (mg/mL)	inactivated pathogen	reference
			bacteria	
kraft lignin	DMSO	15	*Escherichia coli*, *Staphylococcus aureus*, *Pseudomonas aeruginosa*, *Salmonella enteritidis*, *Bacillus cereus*	(81)
pyrolytic lignin	DMSO	5	*Staphylococcus aureus*, *Escherichia coli*	(82)
kraft lignin	Bacto Tryptic Soy Broth	100	*Listeria monocytogenes*, *Staphylococcus aureus*	(83)
			fungi	
organosolv/kraft lignin	DMSO	1–20	*Aspergillus niger*	(84)
organosolv lignin	DMSO	0.48–0.025	*Candida parapsilosis*, *Candida krusei*, *Candida guilliermondii*, *Candida albicans*, *Aspergillus flavus*, *Aspergillus furmigatus*	(85)
organosolv lignin	DMSO	0.5, 5, 10	*Aspergillus niger*, *Saccharomyces cerevisiae*	(86)
			viruses	
lignin–carbohydrate complex	1% H_2_SO_4_ + organic solvents	0.05	encephalomyocarditis virus (EMV)	(87)
	DMEM	0.5	herpes simplex virus (HSV)	(88)
	H_2_O	0.1, 2	EMV, HSV	(89)
lignosulfonate	PBS	10	human immunodeficiency virus (HIV), HSV	(90)
	cell culture medium	70 nM–236.6 μM^a^	HIV	(91)
	cell culture medium	0–0.2	HIV	(92)
	cell culture medium	0–0.5	HIV, HSV	(93)

a In the article referenced, lignosulfonate concentration was expressed as the molar concentration of polymer.

The antibacterial activity of lignin is generally attributed to the phenolic hydroxyl groups, which are able to damage the bacterial cell membrane and lead to the bacteria lysis.^94,95^ The antibacterial activity of phenols and polyphenols is generally known, but the precise mechanism of action is still unclear. The antibacterial performance varies with and depends on the type of lignin and the bacterial strain. For instance, Dong et al. investigated and described a kraft lignin isolated from corn, which was able to efficiently inactivate *Listeria monocytogenes* and *Staphylococcus aureus*, two Gram-positive bacteria, but not Gram-negative bacteria or bacteriophages.^83^ In another study, Lourençon et al. reported that a kraft lignin extracted from eucalyptus can successfully inactivate both Gram-positive bacteria, such as *Bacillus cereus*, *Staphylococcus aureus*, and *Pseudomonas aeruginosa*, as well as Gram-negative bacteria, such as *Escherichia coli* and *Salmonella enteritidis.*(81) In addition to being used as a pure antibacterial agent, lignin can also be blended with or incorporated into more complex systems. A very interesting example was provided by Ritcher et al., who placed lignin around a silver nanoparticle core to achieve excellent antibacterial performance against *Staphylococcus aureus* and *Escherichia coli* without production of environmentally adverse silver ions.^96^ Some studies have also examined the antibacterial activity of polymer films where lignin was used as a filler, prepared analogously to the polymer blends described in the previous paragraph, which showed successful inactivation of various bacteria.^97−99^

Lignin can also inhibit specific species of fungi. The mechanism of fungal inhibition is currently unknown, but is dependent both on the lignin source and extraction process. Gordobil et al. compared the antifungal activity of lignin extracted from both eucalyptus and spruce via organosolv and kraft processes against *Aspergillus niger* and verified that the kraft lignin from eucalyptus exhibited the best antifungal performance.^84^ Another example that is worth mentioning was provided by de Melo et al., who tested a lignin isolated from *Caesalpinia pulcherrima* leaves against a wide number of fungi. The outcome of this study was that a very different amount of the same lignin type can be necessary to inhibit different fungi species.^85^

The antiviral activity of lignin–carbohydrate complex and lignosulfonate, both of which are water-soluble, has been studied in cell culture medium and aqueous solution against a number of viruses. Even though some research groups have tried to establish a relationship between the lignin structure and the antiviral effect, the well-defined antiviral mechanism has not been clarified yet.

Inside the plant wall, lignin is covalently bound to carbohydrates and forms a lignin–carbohydrate complex, which can be extracted from biomass via different methods, such as acidolysis, fractionation, and enzymatic hydrolysis.^100,101^ Lignin–carbohydrate complexes have shown efficient inactivation of encephalomyocarditis virus (EMV) and herpes simplex virus (HSV).^87−89^ Their antiviral activity in aqueous solution was attributed to the inhibition of viral binding and penetration into the host cells. The specific role of lignin in the antiviral activity of the lignin–carbohydrate complex remains unclear.

Lignosulfonate, the only water-soluble technical lignin type, has shown antiviral activity against HSV and human immunodeficiency virus (HIV). The antiviral activity of lignosulfonates was attributed to the structural similarity with heparan sulfate, a proteoglycan found in the proximity of the cell wall where viruses can typically interact with cells. The antiviral mechanism was not completely clarified but was proved to be influenced by sulfur content, molecular weight, and counterion (Na^+^, Ca^2+^, NH_4_^+^).^90−93^

### Challenges and Future Perspectives for the Application of Lignin as Antimicrobial Agent

Lignin has been proven to be an efficient agent for the inhibition of bacteria, fungi, and viruses. The main challenge for the application of lignin as an antimicrobial compound is its heterogeneity in terms of structure, reactive group content, and impurities. Since lignin can be obtained from different natural sources by using various methods, its properties and activity against pathogens can drastically vary. A fundamental mechanistic understanding of the deactivation of bacteria, fungi, and viruses by lignin is, hence, required in order to define a structure–activity dependency profile. Although lignin can be degraded in the environment by specific fungi, bacteria, and enzymes, the fate of this polymer inside the human body is still under debate. Moreover, despite a large number of studies on the biocompatibility of lignin, the consequences of lignin use for biomedical purposes on cells and genes are still mainly unknown and will require a detailed investigation. Regarding the studies about viral inactivation, besides the use of lignin as a macromolecule, it is noteworthy to mention that a number of phenol monomers have been identified and extracted from lignin that displayed efficient antiviral activity against encephalomyocarditis virus,^102−104^ which suggests the involvement of phenolic groups in the antiviral activity of lignin. For this application, only water-soluble lignosulfonates and lignin–carbohydrate complexes in solution have been tested. In a recent study, antiviral lignin surface coatings made of water insoluble lignins were prepared, which showed very efficient inactivation of HSV-2 (>99% after 30 min). Particular attention has been focused on the mechanism behind the antiviral activity of these coatings, which turned out to be strongly related to the lignin phenol content.^105^ The COVID-19 outbreak has highlighted the importance of antiviral surfaces. Lignin is a promising material to develop affordable and sustainable antiviral coatings on a large scale, which deserves additional investigation. New methods to prepare resistant coatings on any type of surface, such as spray and brush coatings, should be tested. The adhesive properties of the coating on different types of substrates, such as glass, wood, or plastic, should also be examined in the future.

## 4 Lignin for Agricultural Applications

Lignin has been applied in several fields of agriculture, such as fertilizer, pesticide, and plant growth regulator.^23^ Since lignin directly derives from plants and can be extracted from agricultural residues, such as straw and husk, its use for agricultural applications is very attractive from the sustainability and circular economy points of view. This Perspective specifically focuses on lignin-based fertilizers, a field of primary importance, where this biopolymer has made a significant contribution and has the potential to make further impact

The use of fertilizers is essential to fulfill the continuously growing demand for food, which accompanies the increase of global population. At the moment, 187 million metric tons of fertilizer are applied every year to allow the production of more than three billion metric tons of crops.^106^ In 2015, the United Nations established 17 sustainable development goals to be accomplished by 2030, including eradicating hunger^107^ and making agriculture sustainable.^108^ With 800 million people suffering from hunger nowadays and a growing global population, it is imperative to further increase the efficiency of crop production.^109^ To match this growing demand, technological innovations will be essential to increase the efficiency of fertilization and other agricultural practices, which are at the moment intrinsically inefficient. A significant portion of the applied fertilizers do not reach the targeted plant and are lost because of evaporation and wash off in the groundwater.^109−111^ Not only is this a waste of nutrients and energy, but it also is a huge environmental problem, which can lead to water eutrophication and dramatic changes in the ecosystems. The challenge of increasing crop production without compromising the environment can be addressed by better controlling nutrient release into the soil using slow- or controlled-release fertilizers.^112−114^ Among the starting materials used for the development of such fertilizers, lignin is very attractive because of its biocompatibility and wide availability at low cost.^115^ Moreover, lignin has many reactive groups that allow the chemical binding of a wide number of nutrient containing groups,^21,116^ which can then be gradually released into the soil upon the biodegradation of lignin.^115^ Several reviews have been published on this topic, which highlight the opportunities for lignin to contribute toward more sustainable agricultural practices (Table 5). Overall, these review articles point out the high potential of lignin to produce agrochemicals with improved efficiency in nutrient release. However, the structural complexity and heterogeneity of lignin always require an elaborated characterization of both reagents and products. These reviews also highlight the lack of a uniform and standardized evaluation of the produced fertilizers.

**Table 5:** Overview of Selected Review Papers on Lignin-Based Fertilizers

review title	publication year	reference
Application of lignin in preparation of slow-release fertilizer: Current status and future perspectives	2022	(117)
Lignin-based controlled release fertilizers: A review	2022	(118)
Novel fertilizing products from lignin and its derivatives to enhance plant development and increase the sustainability of crop production	2022	(119)
Can lignin be transformed into agrochemicals? Recent advances in the agricultural applications of lignin	2021	(23)
Research Progress in Lignin-Based Slow/Controlled Release Fertilizer	2020	(120)
Lignin in Crop Cultivations and Bioremediation	2005	(121)
Nitrogenous Fertilizers From Lignins - a Review	2002	(122)

**Figure 4:**
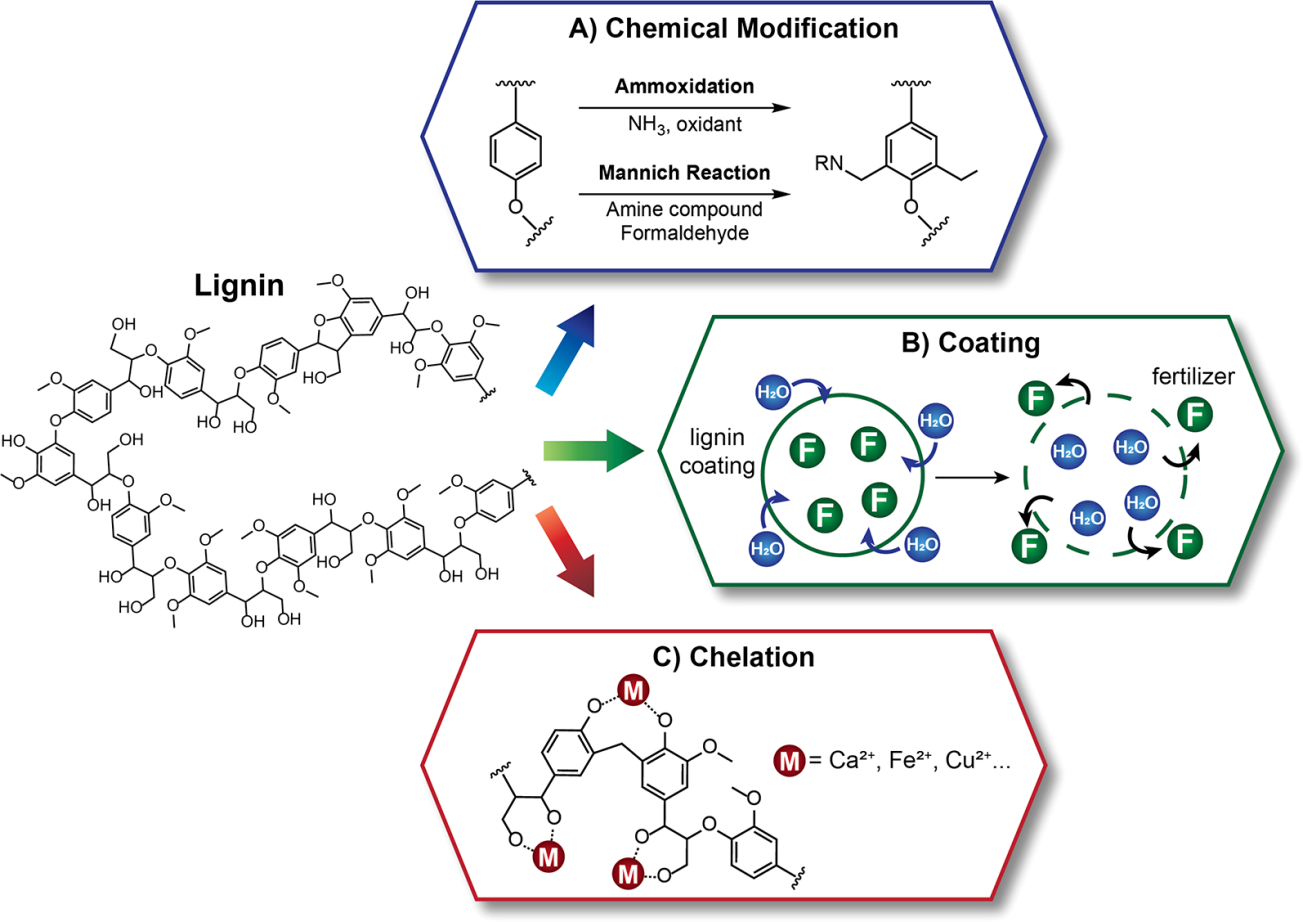
Methods to prepare lignin-based slow-release fertilizers: (A) chemical modification, (B) coating, and (C) chelation.

Lignin-based slow/controlled release fertilizers can be prepared via a number of approaches, where lignin can be (i) modified by chemical reaction and directly constitute the nutrient, (ii) used as coating for the active ingredient, and (iii) applied as a chelating agent for trace element release. These three strategies are illustrated in Figure 4 and summarized in Table 6.

**Table 6:** Preparation of Lignin-Based Slow-Release Fertilizers

preparation method	lignin type	procedure	active ingredient	reference
ammoxidation	kraft lignin	O_2_, 150 °C, 50 min	12 N % content	(123)
	kraft lignin	O_2_, 150 °C, 90 min	13–14 N % content	(124)
	straw pulping solid residue	H_2_O_2_, 90 °C, 90 min	4.9 N % content	(125)
Mannich reaction	soda lignin	formaldehyde, NaOH, 60–80 °C, 3–5 h	5.4–10.2 N % content	(126)
	soda lignin	formaldehyde, ultrasound, 60–90 °C, 3 h	6.9–8.2 N % content	(127)
	soda lignin	formaldehyde, acetic acid, 60 °C, 4 h	3.4–4.2 N % content	(128)
	soda lignin	formaldehyde, NaOH, 60 °C, 3 h	12 N % content	(129)
coating	kraft lignin	drum coating	urea	(130)
	kraft lignin	drum coating	urea	(131)
	acetylated lignosulfonates	fluidized bed	urea	(132)
	soda lignin	turning pan coater	urea	(133)
chelation	kraft lignin	precipitation	Ca^2+^	(134)
	aminated lignin	mixing	Fe^3+^	(135)
	lignosulfonates	mixing	Fe^3+^	(136)
	lignosulfonates	mixing	Fe^3+^	(137)

Most of the lignin-based slow-release fertilizers are prepared by chemically binding nutrients to the reactive groups of lignin. In particular, nitrogen-containing groups are mainly attached to lignin via ammoxidation and Mannich reactions, as shown in Figure 4A.

Ammoxidation involves the oxidation of an organic compound using an oxidant (e.g., O_2_, H_2_O_2_, or H_2_SO_4_) in the presence of ammonia.^122^ The amount of nitrogen that can be incorporated into lignin depends on the reaction conditions, which include temperature, pressure, time, and the type of oxidant.^120^ A nitrogen content of 13–14% was achieved using optimal conditions.^124^ Although this is a well-established method for the preparation of nitrogen-bearing lignin, some drawbacks still need to be addressed, such as frequent damage of the equipment under the required harsh conditions and the easy leakage of ammonia.^117^

The Mannich reaction allows the attachment of amine-group-bearing molecules to lignin in the presence of formaldehyde and can be performed in basic, neutral, or acidic conditions. This reaction modifies the aromatic rings of lignin, in particular the *ortho* and *para* position of the phenols,^126^ and displays higher yields on lower-molecular-weight lignins. To increase the efficiency of the reaction, lignin can be pretreated via phenolation or mild depolymerization.^138^ Although this reaction is very efficient and straightforward, the use of formaldehyde is a serious drawback from a sustainability point of view. A more environmentally friendly alternative should be considered in the future.

Thanks to its aromatic structure and hydrophobic nature, the incorporation of a fertilizer inside a lignin coating can reduce leaching in the environment and groundwater (see Figure 4B). A number of papers have been published on urea incorporation inside lignin coatings prepared by mixing urea and lignin with a sealing agent, such as paraffin. For this process, lignin can be used as is or as previously modified. The nutrient inside the lignin coating can then be released by a rupture mechanism, which means that water vapor enters the coating and dissolves the fertilizers, thereby increasing the osmotic pressure and breaking the coating. If the coating can resist the osmotic pressure increase, the nutrient is instead released via diffusion, which relies on the different concentration of the nutrient inside and outside the coating.^139−141^

The elements necessary for the plant growth are in total 14, divided into major elements (N, P, K, Ca, Mg, and S) and trace elements (Cl, B, Fe, Mn, Cu, Zn, Ni, and Mo).^142,143^ Thanks to the numerous hydroxyl and carbonyl groups in the lignin structure, this polymer can be used to chelate a number of ions for the preparation of trace element fertilizers (see Figure 4C). The ability to create chelating bonds with metal ions depends both on the lignin type and on the metal.^144^

### Challenges and Future Perspectives for the Application of Lignin as Fertilizer

Lignin is a very attractive material for the preparation of slow-release fertilizers because of its biodegradability, low cost, and biocompatibility; hence, a large number of articles have been published on this topic. However, several drawbacks currently limit the application of lignin as fertilizer. For the chemically modified lignin-based fertilizers, the ammoxidation process should be optimized in terms of temperature, pressure and choice of oxidant in order to increase the N % content in the final product. The recycling of ammonia should also be considered. Regarding the Mannich reaction and chelation reaction, additional research work should be invested to use sustainable reagents and avoid the generation of toxic byproducts. Considering the lignin-based slow-release fertilizers prepared via the coating method, the main limitation is that the coating can often be uneven and present cracks, thereby making the nutrient release less controllable than the products prepared via chemical modification. Improvements are, thus, needed to optimize the coating process and quality in order to achieve a more stable slow-release effect. Another implementation could be made in regard to the delivered nutrient type. Overall, most of the developed lignin-based fertilizers bear nitrogen, while the literature regarding fertilizers containing phosphorus, the second most limiting nutrient in soil, is very limited.^145−147^ To achieve this, lignin could be simply phosphorylated,^148^ or phosphorus cross-linkers and phosphorus-containing compounds could be easily incorporated into a lignin carrier.^149^ Another interesting perspective is the development of nanosized lignin-based fertilizers. Nanofertilizers are known to present many advantages over conventional fertilizers,^109,150−152^ and lignin nanoparticles can be prepared with a wide number of methods.^153−156^ Finally, the slow- and controlled-release fertilizers developed so far simply slow down the nutrient distribution in the soil, independent of proximity to the target plant. A more efficient approach to diminish the waste of nutrients in the environment would be to develop plant growth synchronized-release fertilizers that are able to deliver the active principle only in the presence of the plant roots.

## 5 Conclusions and Perspectives

This Perspective has discussed the state-of-the-art methods and opportunities for the valorization of lignin for food packaging, antimicrobial, and agricultural applications. Lignin has the potential to be used in a range of applications, but the use of this biopolymer can be challenging because of a number of problems. Overall, the main complication is the structural and compositional heterogeneity of lignin, which depends on the plant source and extraction process and requires an elaborate characterization of both the starting reagents and products. A consequence of the wide diversity in lignin types is that a well-defined structure–activity dependency should be established for most of the applications where this biopolymer is employed.

For food packaging applications, lignin can be used as a green additive not only to improve the mechanical and gas barrier properties of polymer films but also to provide antioxidant and anti-UV activity. The main challenge for the incorporation of lignin into a polymer film is its compatibility with the surrounding matrix. To avoid heterogeneity and phase separation, particular attention must be placed on the functionalization of the lignin or the lignin nanoparticles in order to improve their compatibility with and dispersion inside the polymer film. A deeper understanding of the interactions between lignin and the packaged products, as well as the digestibility of the film, is also required. Since lignin is hardly degradable in composting conditions, additional attention should also be focused on evaluating the effect of the lignin incorporation on the compostability and degradability of the final product.

Regarding the use of lignin as an antimicrobial agent, the precise mechanism of the interaction between lignin and bacteria, fungi, and viruses is currently still unclear and under debate. The heterogeneity of lignin in terms of molecular weight, impurities, and reactive group content opens the door to many applications in medicine and biology but also complicates the assessment of its activity and safety for the human body. Despite a number of studies that have investigated and demonstrated the antimicrobial activity of lignin in solution, only very limited efforts have been made to use lignin as a coating material to develop antimicrobial surfaces. Since viruses and bacteria can transmit via contact with contaminated surfaces, and the systematic disinfection of surfaces is labor- and time-consuming, the development of coatings that are able to directly inactivate microbes is very useful. Lignin coatings can be easily prepared and tested against bacteria, fungi, and viruses. Additional research work should be focused both on the preparation of such coatings and on their performance against a large spectrum of pathogens.

The last part of this Perspective has discussed the use of lignin for agricultural applications and, in particular, as a fertilizer. The current use of fertilizers is very inefficient. Lignin is a promising starting material for the preparation of controlled-release fertilizers, but some drawbacks need to be overcome to allow their large-scale use. On the one hand, the methods to prepare lignin-based fertilizers require improvement. Ammoxidation and Mannich reactions are both efficient to enrich lignin with nitrogen, but their sustainability should be optimized in terms of reagents, side products, and working conditions. The same also applies to the use of lignin as a coating material to develop controlled-release fertilizers or as depots for the release of essential elements. On the other hand, lignin could be used to design new types of fertilizers. An interesting option to be examined is the use of lignin nanoparticles, and of lignin modified with types of nutrients other than nitrogen, for instance, by modification with or through the incorporation of phosphorus. The development of lignin-based nanofertilizers is another opportunity to implement the yield of crop production. In particular, a promising perspective is to develop systems that would allow the release of nutrients specifically in the proximity of the plant, thus avoiding a waste of nutrients and helping to prevent or reduce environmental pollution.

Overall, the aim of this Perspective was to highlight the potential of lignin, an underutilized natural source that holds a lot of promise not only for food packaging, antimicrobial, and agricultural applications, but also for a range of other technological challenges that call for sustainable materials solutions.
